# SPP1 is a prognostic related biomarker and correlated with tumor-infiltrating immune cells in ovarian cancer

**DOI:** 10.1186/s12885-022-10485-8

**Published:** 2022-12-30

**Authors:** Wen Gao, Dongli Liu, Haiyan Sun, Zhuyan Shao, Peipei Shi, Tingting Li, Sheng Yin, Tao Zhu

**Affiliations:** 1 The Cancer Hospital of the University of Chinese Academy of Sciences (Zhejiang Cancer Hospital), Institute of Basic Medicine and Cancer (IBMC), Chinese Academy of Sciences, Hangzhou, 310022 Zhejiang China; 2 Department of Radiation Oncology, Shanghai General Hospital, Shanghai Jiao Tong University School of Medicine, 200080 Shanghai, P. R. China; 3 Department of Obstetrics and Gynecology, Zhongshan Hospital, Fudan University, 180 Fenglin Road, Shanghai, 200032 China

**Keywords:** SPP1, Ovarian cancer, Prognostic, Tumor immune infiltration, Database mining

## Abstract

**Background:**

Secreted phosphoprotein 1 (SPP1) plays a vital role in tumor progression of multiple cancer types However, it still awaits further exploration whether SPP1 is a bystander or an actual player in the modulation of immune infiltration in ovarian cancer.

**Methods:**

In this study, the expression level of SPP1 was identified by Oncomine, GEPIA and TIMER databases, and the result of SPP1 immumohistochemical staining was acquired by The HPA database. The impact of SPP1 expression level on the clinical outcome of ovarian cancer patients were evaluated via Kaplan–Meier Plotter and PrognoScan dataset. Immune infiltration analyses were conducted using TIMER and TISIDB dataset. In addition, Functional enrichment analyses were performed with Metascape and GeneMANIA database. To verify these findings from the public database, the results were validated in a cohort of ovarian cancer patients.

**Results:**

SPP1 was found to be overexpressed in ovarian tumor tissues and high SPP1 expression was correlated with shorter survivals. Notably, SPP1 expression was positively correlated with infiltrating levels of CD4 + T cells, CD8 + T cells, macrophages, neutrophils, and dendritic cells. Furthermore, SPP1 expression level showed strong correlation with diverse immune cells in ovarian cancer. Of note, functional enrichment analysis suggested that SPP1 was strongly correlated with immune response.

**Conclusions:**

These findings imply that SPP1 is correlated with prognosis and immune cell infiltrating, offering a new potential immunotherapeutic target in ovarian cancer.

**Trial registration:**

Not applicable.

**Supplementary Information:**

The online version contains supplementary material available at 10.1186/s12885-022-10485-8.

## Introductions

Ovarian cancer is the most lethal subtype of the gynecologic malignancies worldwide [1–3]. Despite rapid advancement in the treatment of ovarian carcinoma, a majority of patients eventually suffer poor clinical outcome [4]. Therefore, new therapeutic strategies and paradigms are of urgent need for ovarian patients. Nowadays, immunotherapy has attracted great interest based on the immune regulation of cancer cells. Although immune checkpoint blocking monoclonal antibodies such as PD-1, PD-L1 and CTLA-4 have shown significant prospects in some cancers, the treatment response of ovarian cancer still remains unsatisfied [5, 6]. Previous studies reported that deficiency of infiltrating lymphocytes (TILs) is significantly correlated with worse survival of ovarian cancer patients [7]. The underlying mechanisms remains unclear and needs be elucidated.

Secreted phosphoprotein 1 (SPP1), also known as early T-lymphocyte activation 1 or protein Osteopontin (OPN), located on chromosome 4q22.1, is a multifunctional secretory acidic glycoprotein [8]. SPP1 belongs to the small integrin binding ligand N-linked glycoproteins (SIBLINGs) family which specifically bind and activate matrix metalloproteinases (MMPs), and can be secreted by macrohpages, epithelial cells and endothelial cells [9]. Previous studies showed that SPP1 is abnormally highly expressed in lung cancer, gastric cancer, colon cancer, breast cancer and liver cancer [10–12]. It showed that high-expressed SPP1 is related to tumor staging, lymph node invasion, and tumor growth in lung carcinoma [12, 13]. Previous studies have also highlighted the critical role of SPP1 in ovarian cancer [14]. It showed that SPP1 could promote ovarian cancer growth by activating the AKT signaling pathway in nude mice model [15]. Yet, the underlying mechanisms are still unclear and it is urgent to study the depth profile.

SPP1 can regulate the host immune system via upregulating IL-12 and IFNγ in mouse macrophages and NK cells which indicates that SPP1 may act as a potential role in host immunity [16, 17]. SPP1 is upregulated in human glioma-associated macrophages [18]. A recent study reported that SPP1 can mediate macrophage polarization and facilitate immune escape by upregulating PD-L1 in lung adenocarcinoma [19]. It is reported that SPP1 knockdown could regulate M2 macrophage polarization via upregulating insulin-like growth factor 1 and leukemia inhibitory factor [20]. However, the molecular mechanisms of SPP1 by modulating immune infiltration cell and prognosis of ovarian cancer were still not fully elucidated.

In our present study, we comprehensively assessed SPP1 expression and its correlation with prognostic value of cancer patients in databases including Oncomine, GEPIA, TIMER, PrognoScan, HPA and Kaplan–Meier plotter. We analyzed the association of SPP1 with tumor infiltration immune cells in the ovarian cancer microenvironments via TIMER and TISIDB. Moreover, functional enrichment analysis suggested that SPP1 was strongly correlated with immune response. Our findings in this report highlight the vital role of SPP1 in ovarian cancers and further offer a probable relationship and underlying mechanisms between SPP1 and tumor-immune interactions.

## Materials and methods

### Oncomine database analysis

The expression levels of SPP1 in various cancers were analyzed using the ONCOMINE database (www.oncomine.org) [21, 22]. The cutoff p-value and fold-change values were as follows: *P*-value: 1E-4; fold change:2.0; gene rank: 10%.

### Kaplan–meier plotter analysis

The clinical outcomes between SPP1 expression and ovarian cancer patients were evaluated with Kaplan–Meier Plotter (www.kmplot.com) [23]. Gene symbol of SPP1 is 209875_s_at in Start KM Plotter for 1656 ovarian cancer patients. In pan-cancer plotter for ovarian cancer, 374 patients were included in this study. The overall survival (OS) and Progression Free Survival (PFS) of patients with ovarian cancer were determined by dividing the patient samples into two groups based on best cutoff (high vs. low expression). *P*-value < 0.05 was considered a statistical significance.

### PrognoScan database analysis

PrognoScan dataset is a new database for meta-analysis of the prognostic value of genes (http://dna00.bio.kyutech.ac.jp/PrognoScan/) [24]. We evaluate the relationship between SPP1 expression and clinical outcomes of ovarian cancer patients using GSE14764 dataset, which was obtained from the PrognoScan database. *P*-value < 0.05 was considered a statistical significance.

### GEPIA database analysis

Gene Expression Profiling Interactive Analysis (GEPIA) (http://gepia2.cancer-pku.cn/#index) is an online database to analyze the RNA sequencing expression data of 9,736 tumors and 8,587 normal samples from the The Cancer Genome Atlas (TCGA) and the Genotype- Tissue Expression (GTEx) database [25]. We use this dataset to evaluate SPP1 expression levels in different cancers and we also evaluate SPP1 expression in different tumor stages of ovarian cancer.

### The human protein atlas database

The SPP1 immumohistochemical (IHC) staining analysis was assessed by The Human Protein Atlas (HPA) database (https://www.proteinatlas.org/) [26]. We evaluated the protein expression in ovarian cancer and normal ovary tissue, separately. The SPP1 antibody was HPA027541.

### Immune infiltration analysis

The expression of SPP1 in ovarian carcinoma and the abundances of B cell, CD8 + T cell, CD4 + T cell, macrophage, neutrophil and dendritic cell were evaluated by TIMER database (https://cistrome.shinyapps.io/timer/) [27]. TISIDB dataset (http://cis.hku.hk/TISIDB/) was used to assess the correlations between SPP1 expression and tumor infiltration lymphocytes (TILs) of ovarian cancer. Spearman correlations between expression of SPP1 and immunoinhibitors across ovarian cancer were also performed by TISIDB dataset [28].

### Functional enrichment analysis

We used the Metascape database (https://metascape.org/gp/index.html#/main/step1) to perform Gene Ontology (GO) and Kyoto Encyclopedia of Genes and Genomes (KEGG) pathways analyses of SPP1 [29]. Terms with a *p*-value < 0.01, a minimum count of 3, and an enrichment factor > 1.5 are collected and grouped into clusters based on their membership similarities. The GeneMANIA project is a biological network integration for gene prioritization and predicting gene function (http://genemania.org/). In this study, protein–protein interaction (PPI) network of SPP1 was analyzed with the GeneMANIA [30].

### Validation of SPP1 in independent cohorts

#### Immunohistochemistry

To further validate the results in the public database, we examined both SPP1 and CD8 expression levels in our 60 formalin-fixed paraffin-embedded (FFPE) primary high grade serous ovarian cancer specimen by immunohistochemical staining. Briefly, the tissue slides were first de-paraffined with xylene, hydrated with graded ethanol, and treated with 1 × citrate antigen repair solution at 100 °C for 30 min. Next, the slices were placed in 3% hydrogen peroxide for 15 min to inactivate endogenous peroxidase activity. Then, the tissues were blocked with 5% bovine serum albumin (BSA) for 1 h at room temperature, and incubated overnight at 4 °C with primary antibody (anti-SPP1, ABCAM, ab214050, 1:2000 dilution; anti-CD8, ABCAM, ab245118, 1:1000). Subsequently, the slides were incubated with the secondary antibody (Goat anti-Rabbit, Dako) at 37 °C for 1 h. Finally, the tissue sections were subjected to antigen detection by DAB solution, and hematoxylin staining of the nuclei were performed.

Positive for CD8 immunohistochemical staining were defined as negative (< 1%), low positive (1–5%) and high positive (≥ 5%) according the density in tumor. The expression levels of SPP1 were determined based on the staining intensity (IS) and the percentage of positively-stained tumor cells (PPC). The staining intensity was classified as 0 (negative), 1 (weak staining), 2 (moderate staining) and 3 (strong staining). The percentage of positively-stained tumor cells was scored as 0 (≤ 10% of positive staining), 1 (10–25%), 2 (25–50%), 3 (50–70%) and 4 (> 75%). The final score was determined by PPC × IS. Patients were divided into SPP1 positive expression (the final score ≥ 4) and negative expression group (the final score < 4). We analyzed the correlations between the SPP1 expression and clinical outcomes of EOC patients in order to further verify their relationships.

### Patients

All the 60 HGSOC patients received primary debulking surgery without neoadjuvant chemotherapy between August 2015 and May 2021 at Zhejiang Cancer Hospital. Patients’ clinical characteristics were abstracted including age at diagnosis, preoperative CA125 value, ascites volume, residual disease, FIGO stage, and Progression-free survival (PFS). PFS was calculated as the time from operation to the diagnosis of the first recurrence or last follow-up, which ever came first. Ethical approval for the study was provided by the independent ethics committee of Zhejiang Cancer Hospital (IRB-2022–380).

### Statistical analysis

Statistical analyses were performed by software SPSS version 19.0 (SPSS Inc, Chicago, IL). All tests were two-sides, and a *p* value of less than 0.05 was considered statistically significant. The Chi-square or Mann–Whitney U test was used to compared the difference of clinical characteristics between two groups. The Kaplan–Meier curves with the log-rank test was used to evaluated the statistical significance of the progression-free survival between different groups.

## Results

### Assessment of SPP1 expression levels in different cancers

**Fig. 1:**
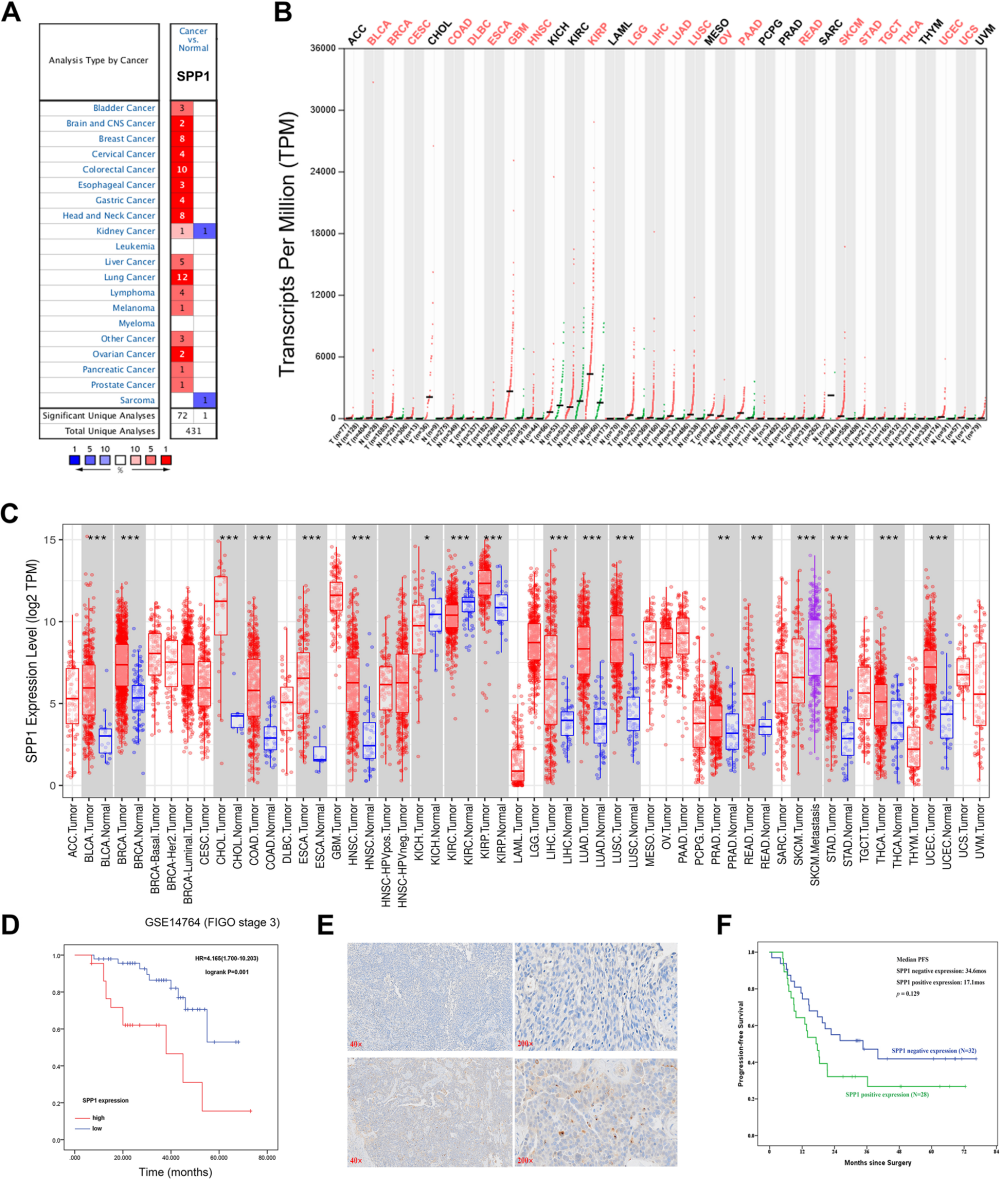
The expression level of SPP1 in different types of tumor tissues and normal tissues. **A** The expression of SPP1 in different types of tumor tissues and normal tissues in the Oncomine database. (*P*-value: 1E-4; fold change:2.0; gene rank: 10%.) **B** The expression of SPP1 in different types of tumor tissues and normal tissues in GEPIA database. **C** The expression of SPP1 in different types of tumor tissues and normal tissues in TIMER database (*P < .05, **P < .01, ***P < .001). **D** Survival analysis of SPP1 expression in stage III disease in independent validation GSE14764 cohort. **E** Representative IHC images of SPP1 negative and positive expression in HGSOC patients. **F** Kaplan–Meier curves of PFS in SPP1 negative and positive expression

In order to explore the SPP1 expression levels in different cancers and the corresponding normal tissues, three different online databases were included in our study. The Oncomine database showed that the expression of SPP1 significantly higher in cancer samples than normal tissues in most datasets (Fig. 1A). The similar results were found in GEPIA database (Fig. 1B). The expression of SPP1 was absolutely up-regulated in bladder cancer (BLCA), brain and CNS cancer, breast cancer (BRCA), cervical cancer (CESC), esophageal cancer (ESCA), gastric cancer (STAD), head and neck cancer (HNSC), liver cancer (LIHC), lung cancer (LUAD and LUSC), lymphoma, melanoma, ovarian cancer (OV), pancreatic cancer (PAAD) and prostate cancer (PRAD) than in corresponding normal tissues. We next evaluate the expression of SPP1 between different tumors and matched normal samples in TIMER dataset with RNA-seq data from TCGA (Fig. 1C). The results are mostly consistent with the above two online databases. However, the expression levels of SPP1 in some cancers were controversial. SPP1 was significantly downregulated in kidney renal papillary cell carcinoma (KIRP), kidney renal clear cell carcinoma (KIRC), kidney chromophobe (KICH) and Skin Cutaneous Melanoma (SKCM) than in control normal samples in TIMER database (Fig. 1C). Taken together, these results demonstrated that SPP1 was up-regulated in multiple cancers suggested that SPP1 may play a crucial biological role in tumor progression.

### Elevated expression of SPP1 correlated with poor outcomes of ovarian cancer

We first evaluated the expression of SPP1 in ovarian cancer and normal ovary tissue using GEPIA database. The results showed that SPP1 was significantly overexpressed in ovarian carcinoma (Supplementary Fig. 1A). In addition, we examined the expression of SPP1 using IHC via HPA database. We found that SPP1 existed in both cell cytoplasm and membrane, and about 35.3% (132/373) ovarian cancer patients with SPP1 high expression. However, the expression of SPP1 was not detected in normal ovary tissue (Supplementary Fig. 1B-1C).

We next investigated the correlation between SPP1 expression and clinical outcomes of ovarian cancer. We observed that stronger SPP1 expression was correlated with overall survival (OS), progression free survival (PFS) and with histology, stage, grade, debulk and chemotherapy (Table 1). Furthermore, we verified the results in independent validation cohort GSE14764 dataset. We found that high expression of SPP1was 29% (23/80) in this database and SPP1 ^*high*^ patients were correlated with poor prognosis in stage III disease consistent with our previous results (Fig. 1D).

**Table 1:** Correlation between SPP1 expression and clinicopathological factors in ovarian cancer by Kaplan–Meier plotter database

Clinicopathological characteristics	Progression free survival (*N* = 1435)					Overall survival (*N* = 1656)				
	N	SPP1 expression		Hazard ratio	*P* value	N	SPP1 expression		Hazard ratio	*P* value
		*low*	*high*				*low*	*high*		
**Histology**										
Serous	1104	553	551	1.15(1–1.33)	0.057	1207	537	670	1.29(1.1–1.5)	***0.0014***
Endometrioid	51	12	39	0.43(0.16–1.15)	0.083	37	27	10	7.14(1.19–42.91)	***0.012***
**Stage**										
1	96	23	73	0.31(0.11–0.88)	0.02	74	55	19	3.04(0.94–9.81)	0.052
2	67	40	27	1.4(0.71–2.75)	0.33	61	23	38	1.59(0.5–5.09)	0.43
3	919	467	452	1.18(1.01–1.37)	***0.036***	1044	512	532	1.34(1.14–1.58)	***0.00042***
4	162	94	68	1.25(0.86–1.83)	0.24	176	106	70	1.21(0.84–1.75)	0.31
**Grade**										
1	37	24	13	4.81(1.47–15.73)	***0.0041***	56	16	40	0.6(0.23–1.58)	0.29
2	256	86	170	0.87(0.64–1.17)	0.35	324	159	165	1.41(1.04–1.92)	***0.026***
3	837	414	423	1.21(1.03–1.43)	***0.023***	1015	452	563	1.25(1.06–1.48)	***0.0078***
**Debulk**										
Optimal	175	44	131	1.5(0.96–2.33)	0.075	189	51	138	1.72(1.07–2.77)	***0.024***
Suboptimal	92	68	24	0.79(0.44–1.43)	0.43	104	48	56	1.69(1.06–2.69)	***0.027***
**Chemotherapy**										
contains platin	305	191	114	1.28(0.96–1.71)	0.088	311	100	211	1.44(1.05–2)	***0.025***
contains Taxol	140	99	41	0.76(0.48–1.19)	0.23	137	49	88	2.17(1.28–3.69)	***0.0034***
contains Taxol + platin	134	38	96	0.76(0.49–1.18)	0.22	131	47	84	2.21(1.28–3.81)	***0.0036***
contains Gemcitabine	51	13	38	0.61(0.31–1.22)	0.16	52	30	22	0.66(0.34–1.28)	0.21

To further validate the results from the public database, immunohistochemical staining was applied to validate the expression of SPP1 in ovarian tissue of HGSOC patient. As shown in Fig. 1E, SPP1 was mainly stained at cytoplasmic, and also with weak membrane staining in the tumor, while negative staining in tumor stromal, which was in consistent with HPA database. SPP1 expression was negative in 32 (53.3%) patients and positive in 28 (46.7%) patients, respectively. By analyzing the correlation between SPP1 expression and patients’ clinical characteristics, we found that there was no significant difference between the two groups in age, FIGO stage, volume of ascites, bowel mesenteric metastasis, lymph node metastasis and residual disease. Patients with SPP1 positive expression showed higher preoperative CA125 value (*p* = 0.032) when compared with SPP negative expression (Supplementary Table 1). The Kaplan–Meier curves shown in Fig. 1F indicated that patients with SPP1 positive expression had a relatively shorter PFS than patients with SPP1 negative expression, although a *p* value of > 0.05.

Taken together, all the above results implied that SPP1 is an important prognostic factor of ovarian cancer.

### SPP1 expression is correlated with infiltrating immune cells in ovarian cancer

**Fig. 2:**
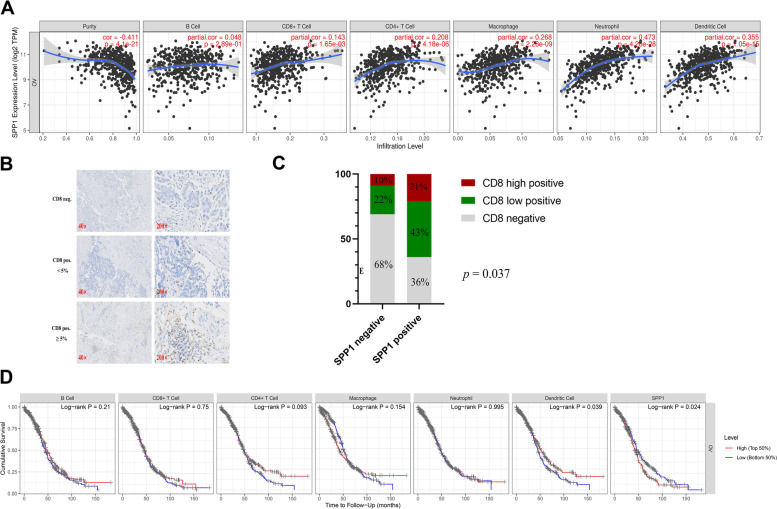
SPP1 expression is correlated with the level of immune infiltration in ovarian cancer. **A** SPP1 expression is correlated with the level of immune infiltration in ovarian cancer. **B** Representative IHC images of CD8 negative, low and high positive expression in HGSOC patients. **C** Correlation of SPP1 and CD8 expression in ovarian cancer tissue. **D** Kaplan–Meier plots of immune infiltration and SPP1 expression levels in ovarian cancer

Since tumor tissues have a mixture of tumor and nontumor, especially tumor-infiltrating lymphocytes, and previous studies showed that tumor survival could be predicted by tumor purity and tumor-infiltrating lymphocytes (TILs) [31–34], we further investigated the relationship between SPP1 expression and tumor purity and TIL abundance via TIMER database and TISIDB database. We found that the expression of SPP1 was significantly correlated with tumor purity of 13 cancer types (*p* < 0.05) (Supplementary Fig. 2). Moreover, SPP1 expression was significantly related with the levels of infiltrating CD8 + cells, CD4 + cells, macrophages, neutrophils and dendritic cells in different types of cancers (Fig. 2A and Supplementary Fig. 2). In ovarian cancer, we observed that SPP1 was significantly negatively associated with tumor purity (*r* = -0.411, *p* = 4.1e-21) but positively correlated with the levels of infiltrating CD8 + cells (*r* = 0.143, *p* = 1.65e-03), CD4 + cells (*r* = 0.208, *p* = 4.18e-06), macrophages (*r* = 0.268, *p* = 2.28e-09), neutrophils (*r* = 0.473, *p* = 4.28–28) and dendritic cells (*r* = 0.355, *p* = 1.05e-15), among which, the correlation with neutrophils and dendritic cells having the most prominent relevancy. However, there was no significant correlation with the level of infiltrating B cells (*r* = 0.048, *p* = 2.89e-01) in ovarian carcinoma (Fig. 2A).

To verify these results, we next analyzed the relationship between SPP1 and CD8 expression in our validation cohorts. As shown in Fig. 2B, among the 60 high-grade serous ovarian cancer patients, CD8 expression was negative, low positive and high positive in 32 (53.3%), 19 (31.7%) and 9 (15.0%) patients, respectively. SPP1 positive expression was correlated with higher CD8 expression (*p* = 0.037) (As shown in Fig. 2C).

We then detected the correlation between infiltrating cell and SPP1 expression by Kaplan–Meier plots using TIMER database. We observed that dendritic cell infiltration (*p* = 0.039) and SPP1 expression (*p* = 0.024) were significantly related to the prognosis of ovarian cancer (Fig. 2D).

Next, we evaluated the associations between SPP1 expression and immune subtypes in ovarian cancer by TISIDB database. The cells were divided into six immuno-phenotypes C1 (wound healing), C2 (IFN-gamma dominant), C3 (inflammatory), C4 (lymphocyte depleted), C5 (immunologically quiet), C6 (TGF-β dominant). We found that SPP1 expression was correlated with C1, C2, C3 and C4 in ovarian cancer (*p* = 3.37e-03) (Supplementary Fig. 3A). We further investigated the associations between SPP1 expression and molecular subtypes in ovarian cancer. The results showed that SPP1 expression was significantly correlated with differentiated, immunoreactive, mesenchymal and proliferative (*p* = 5.75e-19) (Supplementary Fig. 3B). All together, these results may suggest that SPP1was correlated with tumor infiltration immune cells in ovarian cancer.

### Correlation analysis between SPP1 and immune marker expression

**Fig. 3:**
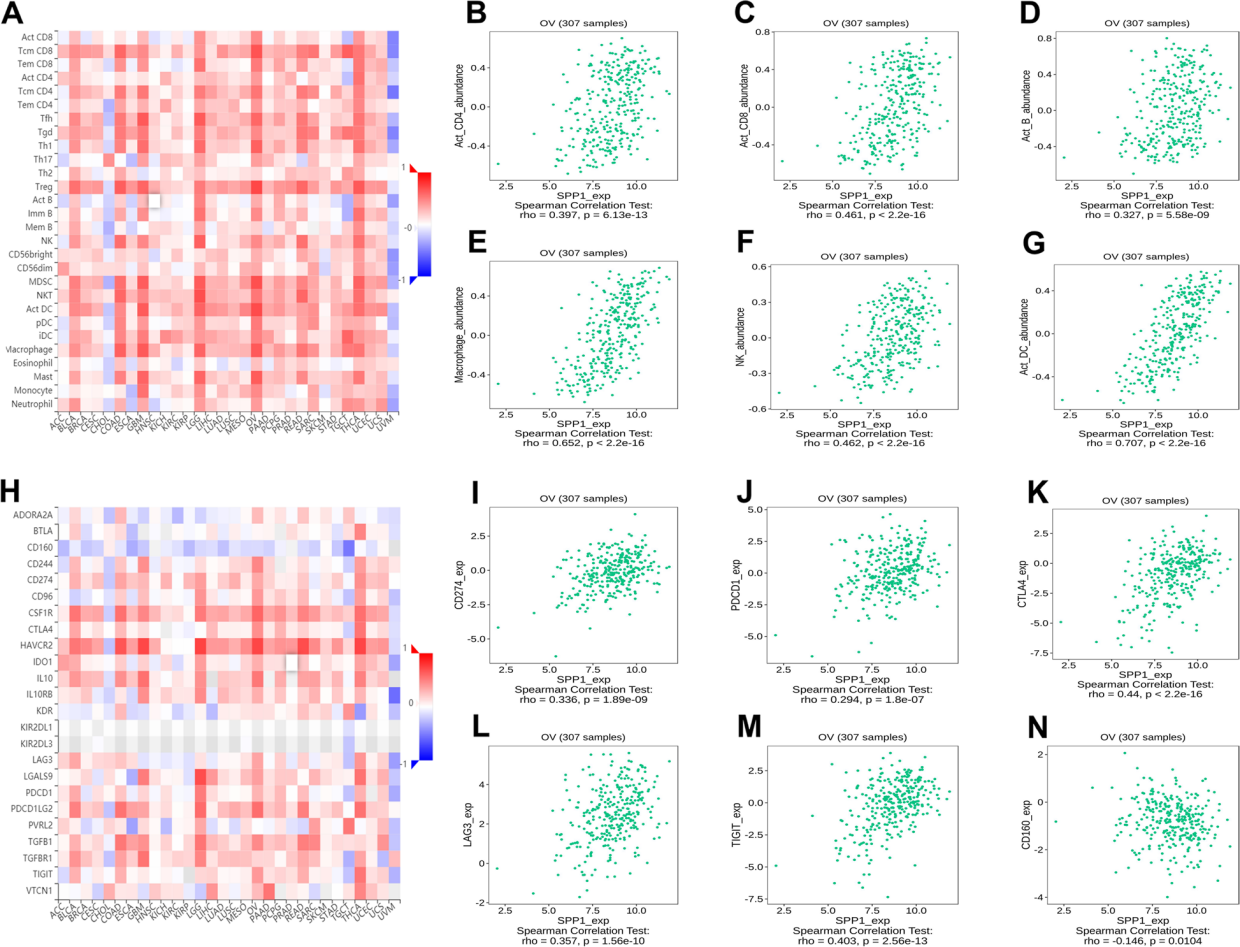
Correlation of SPP1 expression with immune cells in cancer. **A** The landscape of relationship between SPP1 expression and TILs in ovarian cancer (red is positive correlated and blue is negative correlated). **B**–**G** SPP1 expression was positively closely related with infiltrating levels of act_CD4, act_CD8, act_B, macrophage, NK, act_DC. **H** The landscape of relationship between SPP1 expression and immunoinhibitors in ovarian cancer (red is positive correlated and blue is negative correlated). **I**–**N** SPP1 expression was positively closely related with infiltrating levels of CD274, CTLA4, LAG3 and TIGIT, and was negatively correlated with CD160

We next used TIMER and TISIDB online database to further explore the effects of SPP1 expression on tumor infiltration immune cells. The heat map of relationship between SPP1 expression and TILs in different cancers was showed in Fig. 3A. We observed that there was a strong correlation between SPP1 expression and abundance of 28 TILs types in ovarian cancer (Fig. 3B-3G, Supplementary table 2). Next, we assessed the correlations between SPP1 expression and immunoinhibitors of ovarian cancer in TISIDB. The results showed that a significant positive correlation between SPP1 expression and immunoinhibitors, such as CD274 (PD-L1), CTLA4, LAG3 and TIGIT (Fig. 3H-3N, Supplementary table 3), suggesting that SPP1 expression were significantly associated with immune-checkpoint and SPP1 may play an important role in immune tolerance of ovarian cancer.

We further investigated the relationship between SPP1 expression and particular cell subsets including CD8 + T cells, general T cells, monocytes, TAM, macrophages, neutrophils, natural killer cells, dendritic cells, Th1 cells, Th2 cells, Tfh cells, Th17 cells, Treg cells and exhaustion T cells. The results were adjusted based on tumor purity. We observed a significant correlation between SPP1 expression and markers of CD8 + T cell (CD8A, CD8B), T cell (CD3D, CD3E, CD2), monocyte (CD86, CD115), TAM (CCL2, IL10), M1 macrophage (IRF5, COX2), M2 macrophage (CD163, VSIG4, MS4A4A), neutrophils (CD11b, CCR7), NK cell (KIR2DL3, KIR2DL4, KIR3DL1, KIR2DS4), DC (HLA-DPB1, HLA-DRA, HLA-DPA1, BCDA-1, CD11c), Th1 (T-bet, STAT4, IFN-γ,TNF-α), Th2 (GATA3, STAT6,STAT5A,IL13), Tfh (BCL6,IL21), Th17 (STAT3,IL17A), Treg (TGFβ, FOXP3, CCR8, STAT5B), T cell exhaustion (PD-1,CTLA4,LAG3,TIM-3,GZMB) in ovarian cancer (Supplementary table 4). These results suggested that SPP1 may participate the regulation of macrophage polarization, DC infiltration and T cell exhaustion. Taken together, these findings indicated that SPP1 expression significantly correlated with immune microenvironment and may promote tumor immune tolerance process.

### Functional enrichment analysis of SPP1 in patients with ovarian cancer

**Fig. 4:**
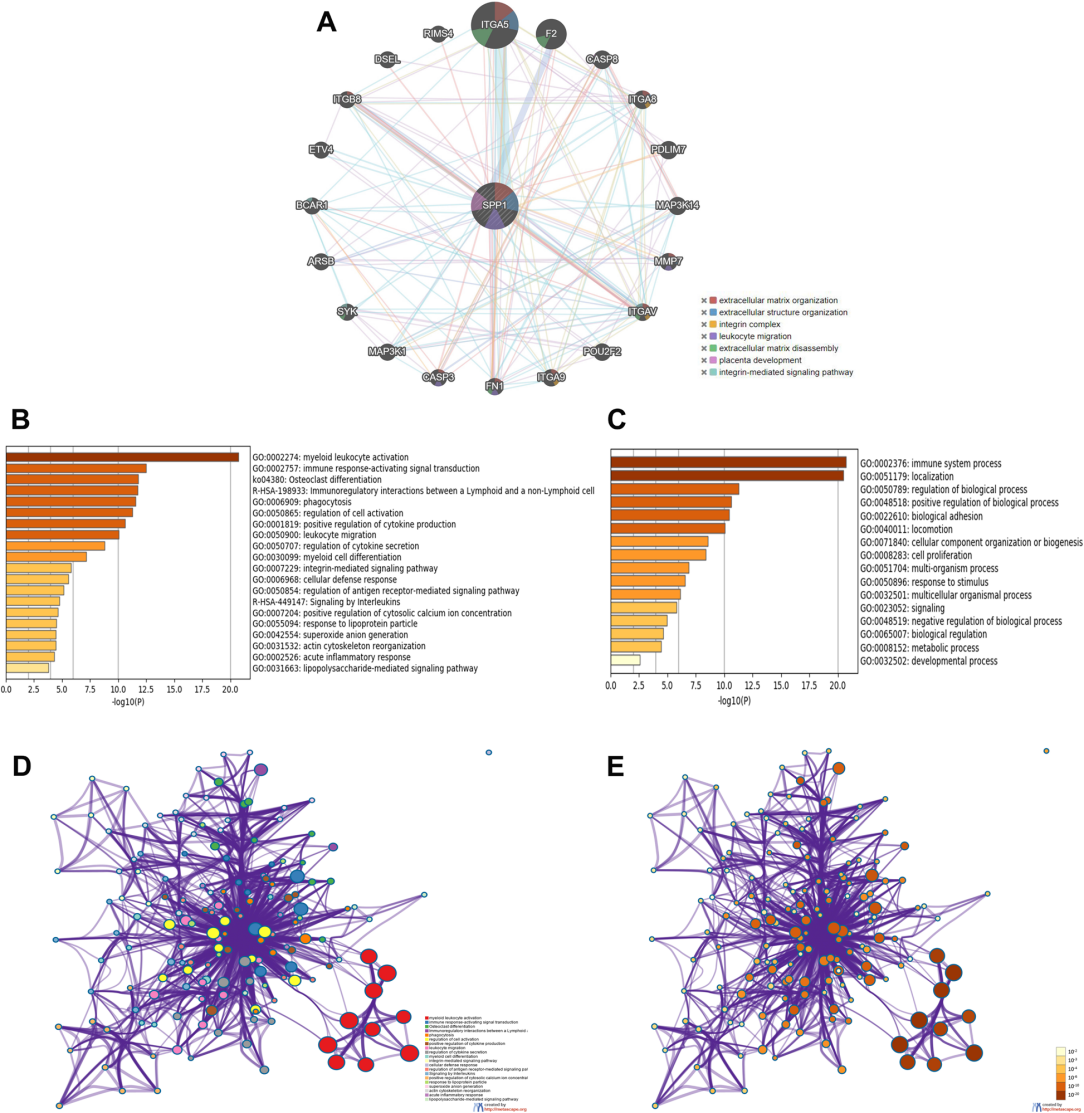
Functional enrichment analysis of SPP1 in patients with ovarian cancer. **A** Protein–protein interaction network of SPP1 networks. The different colors for the network nodes indicate the biological functions of the set of enrichment genes. **B** Bar graph of Gene Ontology (GO) enriched terms colored by p-values. **C** Bar graph of the top level Gene Ontology (GO) biological processes. **D** Network of GO enriched terms colored by cluster ID. **E** Network of GO enriched terms colored by *p*-value

To better understand the interplay functions of SPP1 and their neighboring genes, we analyzed PPI networks using GeneMANIA online dataset. The results showed that the extracellular matrix gene FN1, integrin family gene ITGA5, ITGA8, ITGAV, ITGA9, ITGB8, apoptosis genes CASP3, CASP8, extracellular matrix disassembly gene MMP7, integrin-mediated signaling pathway genes MAP3K1, MAP3K14, leukocyte migration genes PDLIM7, SYK and oncogenes BRCA1, RIMS4, ETV4 and DSEL were closely associated with SPP1 (Fig. 4A). Among them, ITGA5 and F2 were found as the top two significant hallmarks in the PPI network relating to SPP1 (Fig. 4A).

Next, functional enrichment analysis were predicted by analyzing GO and KEGG in Metascape. The top 20 GO enrichment items were classified into three functional groups: 10 items of biological process group, 5 items of molecular function group and 5 items of cellular component group. Consistent with our preceding analysis, the results showed strong relationship with immune response. Top enriched ontology clusters of SPP1 and its neighboring genes included immune response-activating signal transduction, immune system process, immune-regulatory interactions between a lymphoid and a non-lymphoid cell, regulation of cell activation. Moreover, all the pathways achieved from the KEGG analysis were related with immune response (Fig. 4B-4E).

## Discussion

Due to the high invasiveness and migration capabilities, ovarian cancer is one of the leading causes of cancer‐related deaths among the gynecologic malignancies world widely [1]. Therefore, the determination of molecular markers has attracted much attention in the treatment and prognosis of ovarian cancer. SPP1 is a secreted arginine glycine aspartic acid containing phosphorylated glycoprotein overexpressed in various malignant neoplasms and it is reported to be involved in various functions, such as in cell adhesion and migration, apoptosis and bone calcification. SPP1 is often overexpressed in multiple cancers including pancreatic cancer [35], lung cancer [36], gastric cancer, hepatocellular cancer, breast cancer and colon cancer [10]. Previous data have also highlighted the critical role of SPP1 in ovarian cancer [14]. Evidence showed that SPP1 could activate the AKT signaling pathway, and promote ovarian cancer growth in nude mice model[15]. However, the underlying mechanisms are still unclear and need be elucidated.

In recent years, tumor immunotherapy such as anti-PD-1/PD-L1/CTLA-4 monoclonal antibody and chimeric antigen receptor T-cell (CAR-T) immunotherapy has extensively been attentioned as an important part of combined therapy. Immunotherapy is fundamentally different from targeted therapy or chemotherapy [5]. Instead of targeting cancer cells directly, it recruits and activates core immune guardian T cells to recognize and eliminate cancer cells through antigen antibody response [37]. Unfortunately, not every patient responds to immunotherapy, especially in ovarian cancer [38]. Therefore, it is urgent to identify new potential targets for immune-related therapy. Previous studies showed that SPP1 participate immune and inflammatory response [39], and it can further promote cancer invasiveness in inflammatory conditions [35]. To gain more detailed insights into the potential immune functions of SPP1 in ovarian cancer and its regulatory network, we performed the bioinformatics analysis of public data to guide future research in ovarian cancer.

In this study, we analyzed the expression and prognosis of SPP1 in 33 different types of cancers by three different online databases: Oncomine database, GEPIA database and TIMER database. All the results suggested that SPP1 mRNA was upregulated in most carcinoma including ovarian cancer. HPA database indicated that SPP1 protein existed in both cellular membrane and cytoplasm, and about 35.3% ovarian cancer patients with SPP1 high expression, while SPP1 expression was not detected in normal ovary tissue. Kaplan–Meier plotter database found elevated SPP1 was associated with worse outcomes. Our validation cohort indicated that higher expression level of SPP1 was correlated with worse PFS than those with lower SPP1 expression. Although Log-rank test showed a p value of > 0.05 which may because of the low sample size, the survival curves was well-separate between the two groups. The above results together imply that SPP1 may have an important value as an unfavorable prognostic biomarker of ovarian cancer.

Immune infiltrating cells in the tumor microenvironment (TME) have been shown to play a key role in tumor progression and influence clinical outcomes in cancer patients. Single-cell RNA sequencing (scRNA-seq) identified that SPP1-CD44 axis was a unique interaction between macrophages and HCC malignant cells, suggesting the role of macrophage-derived SPP1 in the progress of HCC [40]. Another single-cell and spatial analysis revealed interaction of FAP + fibroblasts and SPP1 + macrophages in colorectal cancer, and their presence is negatively correlated with lymphocyte infiltration and predicted a poor patient survival [41]. In our report, we found that SPP1 expression was correlated with TILs abundance. We demonstrated that SPP1 positively correlated with CD8 + cells, CD4 + cells, macrophages, neutrophils and dendritic cells (Fig. 2A). We further observed that dendritic cell infiltration and SPP1 expression were significantly associated with the prognosis of ovarian cancer, although the prognostics value was completely different (Fig. 2D).

We next observed that SPP1 expression was positively related with immune-checkpoint, such as CD274, CTLA-4, LAG3 and TIGIT (Fig. 3H-3N), suggesting that SPP1 may play an important role in immune tolerance of ovarian cancer. However, our paper only studies the correlation between them, while the specific underlying mechanism needs to be further developed. Furthermore, we found that expression of SPP1 correlated with macrophage and DC infiltration. Many tolerogenic DCs (tolDCs) could bind to PD-1(PDCD1), subsequently promoting tolerance via induction of clonal anergy and Treg differentiation. CTLA-4 can regulate costimulatory molecules expressed by DCs, impairing the priming of naïve T cells [42, 43]. We speculated that SPP1 might initiate immunosuppression or immune escape by recruiting DC cells through the above ways. Taken together, these findings indicated that SPP1 expression significantly correlated with tumor immune microenvironment and may promote tumor immune tolerance process.

Enrichment analysis of target gene sets can help reveal important networks of transcription factors, target genes and pathway hallmarks. Our study suggested that neighboring gene network of SPP1 was associated with extracellular matrix gene FN1, integrin family gene ITGA5, ITGA8, ITGAV, ITGA9 and ITGB8, apoptosis genes CASP3 and CASP8, integrin-mediated signaling pathway genes MAP3K1 and MAP3K14 and leukocyte migration genes PDLIM7 and SYK. We found that ITGA5 was the significant hallmark in the PPI network relating to SPP1. (Fig. 4A). Previous study showed that cancer-associated fibroblasts recruit ITGA5 high HGSOC ascitic tumor cells to form metastatic units, suggesting the potential biologic regulatory mechanism of ITGA5 in ovarian cancer [44]. In our paper, SPP1 mediate the worse outcome of ovarian patients may via ITGA5, and the mechanism related research will be further conducted. Functional enrichment analysis suggested strong relationship with immune response including immune response-activating signal transduction, immune system process, immune-regulatory interactions. (Fig. 4B-4E). The findings further highlighted that SPP1 was closely related with immune response.

In conclusion, SPP1 might be an important regulator of tumor immune cell infiltration and act as a promising prognostic biomarker for ovarian cancer patients, offering a new probable immunotherapeutic target in ovarian cancer.

## Supplementary Information

**Additional file 1:** **Supplementary Figure 1.** The expression level of SPP1 in ovarian cancer in GEPIA. (A) The expression level of SPP1 in ovarian cancer and normal tissues. (B) Representative IHC images of SPP1 expression in ovarian cancer tissue. (C) Representative IHC images of SPP1 expression in normal ovarian tissue. **Supplementary Figure 2.** Correlation of SPP1 expression with tumor-infiltrating immune cells in various types of cancers via the TIMER database. **Supplementary Figure 3.** (A) Correlation of SPP1 expression and immune subtypes (C1: wound healing, C2: IFN-gamma dominant, C3: inflammatory, C4: lymphocyte depleted) in ovarian cancer. (B) Correlation of SPP1 expression and immune subtypes (differentiated, immunoreactive, mesenchymal, proliferative) in ovarian cancer. **Supplementary Table 1.** Correlation between SPP1 expression and patients’ clinical characteristics. **Supplementary Table 2.** Spearman correlations between expression of SPP1 and TILs of ovarian cancer in TISIDB. **Supplementary Table 3.** Spearman correlations between expression of SPP1 and Immunoinhibitors of ovarian cancer in TISIDB. **Supplementary Table 4.** Correlation analysis between SPP1 and relate genes and markers of immune cells in TIMER.

## Data Availability

All data utilized in this study are included in the article and all data are available on reasonable request from the corresponding author. Direct web links of datasets about; Oncomine: (www.oncomine.org); Kaplan–Meier Plotter: www.kmplot.com; Prognoscan: http://dna00.bio.kyutech.ac.jp/PrognoScan/; GEPIA: http://gepia2.cancer-pku.cn/#index; HPA: https://www.proteinatlas.org/; TIMER: https://cistrome.shinyapps.io/timer/; TISIDB: http://cis.hku.hk/TISIDB/; Metascape: https://metascape.org/gp/index.html#/main/step1; GeneMANIA: http://genemania.org/.
