# Supplementary material for: SPP1 is a prognostic related biomarker and correlated with tumor-infiltrating immune cells in ovarian cancer
Source: BMC Cancer. 2022 Dec 30;22:1367. doi: 10.1186/s12885-022-10485-8 (PMC9805166; doi:10.1186/s12885-022-10485-8)
Supplement: Supplementary file 1 — Additional file 1: Supplementary Figure 1. The expression level of SPP1 in ovarian cancer in GEPIA. (A) The expression level of SPP1 in ovarian cancer and normal tissues. (B) Representative IHC images of SPP1 expression in ovarian cancer tissue. (C) Representative IHC images of SPP1 expression in normal ovarian tissue. Supplementary Figure 2. Correlation of SPP1 expression with tumor-infiltrating immune cells in various types of cancers via the TIMER database. Supplementary Figure 3. (A) Correlation of SPP1 expression and immune subtypes (C1: wound healing, C2: IFN-gamma dominant, C3: inflammatory, C4: lymphocyte depleted) in ovarian cancer. (B) Correlation of SPP1 expression and immune subtypes (differentiated, immunoreactive, mesenchymal, proliferative) in ovarian cancer. Supplementary Table 1. Correlation between SPP1 expression and patients’ clinical characteristics. Supplementary Table 2. Spearman correlations between expression of SPP1 and TILs of ovarian cancer in TISIDB. Supplementary Table 3. Spearman correlations between expression of SPP1 and Immunoinhibitors of ovarian cancer in TISIDB. Supplementary Table 4. Correlation analysis between SPP1 and relate genes and markers of immune cells in TIMER. [file 12885_2022_10485_MOESM1_ESM.docx]

**Supplementary Figure 1.** The expression level of SPP1 in ovarian cancer in GEPIA. (A) The expression level of SPP1 in ovarian cancer and normal tissues. (B) Representative IHC images of SPP1 expression in normal ovarian tissue. (C) Representative IHC images of SPP1 expression in ovarian cancer tissue.

**Supplementary Figure 2.** Correlation of SPP1 expression with tumor-infiltrating immune cells in various types of cancers via the TIMER database.

**Supplementary Figure 3**. (A) Correlation of SPP1 expression and immune subtypes (C1: wound healing, C2: IFN-gamma dominant, C3: inflammatory, C4: lymphocyte depleted) in ovarian cancer. (B) Correlation of SPP1 expression and immune subtypes (differentiated, immunoreactive, mesenchymal, proliferative) in ovarian cancer.

**Supplementary Table 1.** Correlation between SPP1 expression and patients’ clinical characteristics

|  | SPP1 expression | |  |
| --- | --- | --- | --- |
|  | Negative (n=32) | Positive (n=28) | *P* value |
| Median age | 52.3 years | 57.0 years | 0.137 |
| FIGO Stage |  |  |  |
| III | 24 (75.0%) | 22 (78.6%) |  |
| IV | 8 (25.0%) | 6 (21.4%) | 0.770 |
| Median preoperative serum CA125 | 1151.7U/ml | 2242.7U/ml | 0.032 |
| Median volume of Ascites | 1000.0 mL | 1000.0 mL | 0.608 |
| Bowel mesenteric metastasis |  |  |  |
| No | 4 (12.5%) | 5 (17.9%) |  |
| Yes | 28 (87.5%) | 23 (82.1%) | 0.721 |
| Lymph node metastasis |  |  |  |
| No | 14 (43.8%) | 15 (53.6%) |  |
| Yes | 18 (56.2%) | 13 (46.4%) | 0.605 |
| Residual disease |  |  |  |
| NGR | 21 (65.6%) | 15 (53.6%) |  |
| Not NGR | 11 (34.4%) | 13 (46.4%) | 0.431 |

FIGO, International Federation of Gynecology and Obstetrics; NGR, no gross residual disease.

**Supplementary Table 2.** Spearman correlations between expression of SPP1 and TILs of ovarian cancer in TISIDB.

| **Variables** | **Cor** | **P value** |
| --- | --- | --- |
| Activated CD8 T cell (Act CD8) | 0.707 | *** |
| Central memory CD8 T cell (Tcm CD8) | 0.676 | *** |
| Effector memory CD8 T cell (Tem CD8) | 0.521 | *** |
| Activated CD4 T cell (Act CD4) | 0.397 | *** |
| Central memory CD4 T cell (Tcm CD4) | 0.544 | *** |
| Effector memory CD4 T cell (Tem CD4) | 0.402 | *** |
| T follicular helper cell (Tfh) | 0.598 | *** |
| Gamma delta T cell (Tgd) | 0.462 | *** |
| Type 1 helper cell (Th1) | 0.619 | *** |
| Type 17 helper cell (Th17) | 0.383 | *** |
| Type 2 helper cell (Th2) | 0.163 | * |
| Regulatory T cell (Treg) | 0.721 | *** |
| Activated B cell (Act_B) | 0.327 | *** |
| Immature B cell (Imm_B) | 0.454 | *** |
| Memory B cell (Mem_B) | 0.193 | ** |
| Natural killer cell (NK) | 0.462 | *** |
| CD56bright natural killer cell (CD56bright) | 0.31 | *** |
| CD56dim natural killer cell (CD56dim) | 0.333 | *** |
| Myeloid derived suppressor cell (MDSC) | 0.685 | *** |
| Natural killer T cell (NKT) | 0.534 | *** |
| Activated dendritic cell (Act_DC) | 0.707 | *** |
| Plasmacytoid dendritic cell (pDC) | 0.52 | *** |
| Immature dendritic cell (iDC) | 0.496 | *** |
| Macrophage (Macrophage) | 0.652 | *** |
| Eosinophil (Eosinophil) | 0.247 | *** |
| Mast cell (Mast) | 0.639 | *** |
| Monocyte (Monocyte) | 0.466 | *** |
| Neutrophil (Neutrophil) | 0.394 | *** |

Cor, R value of Spearman’s correlation; *P < 0.01; **P < 0.001; ***P < 0.0001.

**Supplementary Table 3.** Spearman correlations between expression of SPP1 and Immunoinhibitors of ovarian cancer in TISIDB.

| **Variables** | **Cor** | **P value** |
| --- | --- | --- |
| CD160 | -0.146 | 0.0104 |
| ADORA2A | 0.271 | *** |
| BTLA | 0.031 | 0.587 |
| CD244 | 0.474 | *** |
| CD274 | 0.336 | *** |
| CD96 | 0.403 | *** |
| CSF1R | 0.605 | *** |
| CTLA4 | 0.44 | *** |
| HAVCR2 | 0.727 | *** |
| IDO1 | 0.313 | *** |
| IL10 | 0.531 | *** |
| IL10RB | 0.312 | *** |
| KDR | 0.127 | 0.0263 |
| LAG3 | 0.357 | *** |
| LGALS9 | 0.382 | *** |
| PDCD1 | 0.294 | *** |
| PDCD1LG2 | 0.567 | *** |
| PVRL2 | -0.008 | 0.893 |
| TGFB1 | 0.457 | *** |
| TGFBR1 | 0.165 | * |
| TIGIT | 0.403 | *** |
| VTCN1 | 0.268 | *** |

Cor, R value of Spearman’s correlation; *P < 0.01; **P < 0.001; ***P < 0.0001.

**Supplementary Table 4.** Correlation analysis between SPP1 and relate genes and markers of immune cells in TIMER.

| **Variables** | **Gene markers** | **None** | | **Purity** | |
| --- | --- | --- | --- | --- | --- |
|  |  | **Cor** | **P value** | **Cor** | **P value** |
| CD8+ T cell | CD8A | 0.378 | *** | 0.182 | * |
|  | CD8B | 0.276 | *** | 0.124 | 0.05 |
| T cell (general) | CD3D | 0.442 | *** | 0.215 | ** |
|  | CD3E | 0.431 | *** | 0.202 | * |
|  | CD2 | 0.474 | *** | 0.264 | *** |
| Monocyte | CD86 | 0.681 | *** | 0.56 | *** |
|  | CD115 (CSF1R) | 0.604 | *** | 0.485 | *** |
| TAM | CCL2 | 0.602 | *** | 0.439 | *** |
|  | CD68 | 0.723 | *** | 0.644 | *** |
|  | IL10 | 0.524 | *** | 0.368 | *** |
| M1 Macrophage | INOS(NOS2) | -0.028 | 0.625 | -0.117 | 0.0647 |
|  | IRF5 | 0.279 | *** | 0.196 | * |
|  | COX2(PTGS2) | 0.227 | *** | 0.146 | 0.0211 |
| M2 Macrophage | CD163 | 0.65 | *** | 0.537 | *** |
|  | VSIG4 | 0.704 | *** | 0.613 | *** |
|  | MS4A4A | 0.596 | *** | 0.451 | *** |
| Neutrophils | CD66b(CEACAM8) | -0.109 | 0.0588 | -0.078 | 0.219 |
|  | CD11b(ITGAM) | 0.661 | *** | 0.588 | *** |
|  | CCR7 | 0.328 | *** | 0.137 | 0.0306 |
| Natural killer cell | KIR2DL1 | 0.136 | 0.0175 | 0.046 | 0.474 |
|  | KIR2DL3 | 0.233 | *** | 0.151 | 0.0168 |
|  | KIR2DL4 | 0.368 | *** | 0.195 | * |
|  | KIR3DL1 | 0.216 | ** | 0.123 | 0.0528 |
|  | KIR3DL2 | 0.146 | 0.0112 | 0.029 | 0.644 |
|  | KIR3DL3 | 0.106 | 0.0667 | 0.093 | 0.142 |
|  | KIR2DS4 | 0.197 | ** | 0.103 | 0.106 |
| Dendritic cell | HLA-DPB1 | 0.495 | *** | 0.301 | *** |
|  | HLA-DQB1 | 0.316 | *** | 0.12 | 0.06 |
|  | HLA-DRA | 0.512 | *** | 0.335 | *** |
|  | HLA-DPA1 | 0.49 | *** | 0.303 | *** |
|  | BCDA-1(CD1C) | 0.389 | *** | 0.224 | ** |
|  | CD11c(ITGAX) | 0.64 | *** | 0.516 | *** |
| Th1 | T-bet(TBX21) | 0.407 | *** | 0.209 | ** |
|  | STAT4 | 0.33 | *** | 0.137 | 0.0305 |
|  | STAT1 | 0.136 | 0.0179 | 0.078 | 0.218 |
|  | IFN-γ(IFNG) | 0.335 | *** | 0.15 | 0.0178 |
|  | TNF-α(TNF) | 0.388 | *** | 0.263 | *** |
| Th2 | GATA3 | 0.318 | *** | 0.142 | 0.0252 |
|  | STAT6 | 0.011 | 0.853 | 0.016 | 0.796 |
|  | STAT5A | 0.183 | * | 0.153 | 0.0157 |
|  | IL13 | 0.083 | 0.149 | 0.056 | 0.38 |
| Tfh | BCL6 | -0.019 | 0.742 | 0.035 | 0.588 |
|  | IL21 | 0.086 | 0.137 | 0.102 | 0.11 |
| Th17 | STAT3 | 0.179 | ** | 0.1 | 0.114 |
|  | IL17A | 0.128 | 0.0264 | 0.084 | 0.185 |
| Treg | TGFβ(TGFB1) | 0.446 | *** | 0.3 | *** |
|  | FOXP3 | 0.392 | *** | 0.209 | ** |
|  | CCR8 | 0.38 | *** | 0.245 | *** |
|  | STAT5B | -0.06 | 0.299 | -0.09 | 0.158 |
| T cell exhaustion | PD-1(PDCD1) | 0.301 | *** | 0.131 | 0.0381 |
|  | CTLA4 | 0.455 | *** | 0.237 | ** |
|  | LAG3 | 0.366 | *** | 0.259 | *** |
|  | TIM-3(HAVCR2) | 0.72 | *** | 0.625 | *** |
|  | GZMB | 0.403 | *** | 0.19 | * |

None, correlation without adjustment. Purity, correlation adjusted by purity. *P < 0.01; **P < 0.001; ***P < 0.0001.
